# In Vitro Evaluation of Remineralization Potential of Five Toothpastes on Soft Drink-Eroded Human Enamel and Dentine

**DOI:** 10.7759/cureus.62921

**Published:** 2024-06-22

**Authors:** Mai M Alhamdan, Jonathan C. Knowles, Ailbhe V. McDonald

**Affiliations:** 1 Department of Prosthetic Dental Sciences, King Saud University, Riyadh, SAU; 2 Division of Biomaterials and Tissue Engineering, University College London, London, GBR; 3 Department of Restorative Dentistry, University College London, London, GBR

**Keywords:** dentine groups, remineralization, tooth erosion, carbonated beverages, saliva artificial

## Abstract

Objective: The purpose of this in vitro study was to evaluate the potential remineralization of enamel and dentine erosion lesions after the application of five different toothpastes.

Methodology: A total of 104 enamel and dentine samples were prepared from maxillary third molars. Each group was divided according to the toothpaste application mode (topical = 56; brushing = 48) and the toothpaste used seven topical groups and six brushing groups (n = 8). The groups included negative control (NC), positive control (PC), Sensodyne Pronamel (SP), Regenerate (R), Regenerate with boosting serum (R+), Colgate Duraphat 5000 (CD), and tooth mousse (TM).

Results: The statistical analysis showed significant surface microhardness (SMH) change. All enamel groups showed a significant decrease in SMH compared to NC for both application modes. However, no significance was recorded between test groups. Similar results were observed between dentine groups and their relevant controls for both application modes, except brushed R and R+ groups, which were insignificant to their NC. For topical groups, TM showed a significant increase in SMH. While R and R+ showed lower loss than SP and CD.

Conclusions: All tested agents offered a degree of remineralization in both enamel and dentine with no significant difference between agents in enamel groups while R, R+, and TM offered better results in dentine groups.

Clinical significance: For dentine groups, similar findings were observed with superior tooth surface protection with the application of TM over other agents. Tooth surface remineralization was achieved when agents were either applied topically or brushed over the surface.

## Introduction

Erosion of dental tissues refers to the irreversible chemical and chemical-mechanical processes that involve the dissolution of dental hard tissues by acids with no inclusion of any bacterial action and mostly through external mediums, like diet (soft drinks, energy drinks, fruit juices) [1]. During the past few years, interest in erosion has grown for both clinicians and researchers. Erosion can affect all tooth tissue layers, from enamel to dentin and sometimes cementum. In the early phase of erosion, the enamel is superficially dissolved without clinically detectable changes and dentin becomes affected only at a later stage [2]. In turn, this process ends in dentine hypersensitivity, which represents an increasing problem in dentistry [3]. There is some evidence that the presence of erosion is growing steadily among all age groups with high prevalence in children and adolescents [4]. This was suggested to be related to the recent increased consumption of fruit juices, soft drinks, and power drinks [1]. Cola is one of the most popular acidic drinks with a pH of 2.74, the lowest pH among their tested food and drinks. Its erosive effect on teeth is well documented, causing the largest change in the tooth surface hardness [5]. Repeated exposures to acidic drinks lead to sustained low intraoral pH below the critical pH (pH 5.5) and allow the development of decalcification lesions [6].

The treatment of the basic principle of tooth erosion is to reduce acid consumption and improvement of remineralization [7]. In the last decade, new materials have been developed that may have value for the remineralization of enamel and dentine affected by dental erosion [8]. Various fluoridated toothpaste formulations have been long tested and proved to be successful in reducing the incidence of demineralization by increasing the fluoride content in saliva [9]. The dental breakthrough of bioactive glass remineralization technology is being widely used in various aspects of dentistry. Bioactive glass formulations undergo dissolution when subjected to an aqueous solution changing the solution composition and pH, which in turn releases bioavailable calcium, sodium, and phosphate ions, which are the main contributors in the remineralization process [10]. The casein‑phosphopeptide‑stabilized amorphous-calcium-phosphate complex toothpaste (CPP‑ACP) has been proven to both prevent and reverse demineralization in various in vitro and in vivo studies [11]. It has been proposed that its mode of action involves buffering free calcium and phosphate ions at the tooth surface when ACP localizes there. This state of supersaturation with regard to hydroxyapatite is responsible for the prevention of demineralization and initiates remineralization [12].

As per the author’s knowledge, the remineralization potential of toothpaste has not been studied so far under different application modes (topical application vs. brushing) or their effects compared on different eroded tooth tissues (enamel vs. dentine). The current study has been undertaken to evaluate and compare the remineralization potentials of bioactive glass fluoridated toothpaste (Regenerate™ Enamel Science (Unilever Europe, London, UK) and Regenerate™ with boosting serum (Regenerate™ Enamel Science, Unilever Europe)), CPP‑ACP tooth cream (GC Tooth Mousse - Recaldent^TM^, GC Corporation, Tokyo, Japan), and fluoride-containing toothpaste with different concentrations (Sensodyne Pronamel (GlaxoSmithKline Consumer Healthcare, Surrey, UK) and Colgate Duraphat 5000 (Colgate-Palmolive, New York, NY)) on enamel and dentine erosion produced by a soft drink (Coca-Cola, Atlanta, GA). The effect of the application mode was also examined as toothpaste slurries were either topically applied or brushed onto the surface.

## Materials and methods

In the present study, 208 samples were prepared from extracted maxillary third molars (104 enamel slabs and 104 dentine discs). Sample size calculation was done using G*Power 3.1 (Franz Faul, Germany) based on the results of a preliminary pilot study. The effect size was estimated to be 0.45 on standardized β/α. Finally, the software computed the total sample size of 104 for 13 groups (n = 8) at a significance of p-value < 0.05 with a power of 0.8 using F test sample power analysis.

Freshly extracted sound maxillary and mandibular third molars were collected (NHS Research Ethics Committee (REC) ethical approval no. 11LO/0939). The current study is a human observational study and it has conformed to the Strengthening the Reporting of Observational Studies in Epidemiology (STROBE) guidelines. Soft tissue debris on teeth was cleaned and inspected under a polarized light microscope (magnification x25) for cracks and white spot lesions, followed by disinfection in 5.25% sodium hypochlorite solution for one hour and then kept in 0.1% thymol solution at 4°C until the time of use [8,13].

Specimens of enamel were developed by polishing flat the buccal and lingual surfaces of the crowns of molars while dentine discs were prepared by sectioning the crowns of molars horizontally into discs. Each group was split into two application categories (topical = 56 and brushing = 48). Teeth in the topical category were assigned randomly into seven further groups concerning the treatment received (n = 8). The groups included the following: negative control (NC), unexposed to any product; positive control (PC), received erosive cycles but no remineralization treatment; Sensodyne Pronamel (SP); Regenerate (R); Regenerate with boosting serum (R+); Colgate Duraphat 5000 (CD); and tooth mousse (TM). Teeth in the brushing category were allocated into six groups only including controls and all test agents except TM, as it is recommended for topical application only by the manufacturer. The toothpastes used, including their active ingredients, are listed in Table 1.

**Table 1:** Tested toothpastes and their active ingredients.

Group	Toothpaste	Active ingredients
R	Regenerate^TM^ Enamel Science, NR‑5^TM ^Technology Advanced Toothpaste (Unilever Europe, London, UK)	Calcium silicate (Ca_2_SiO_4_)and sodium phosphate (Na_3_PO_4_) (NR-5 technology) + sodium fluoride (1450ppm NaF)
R+	Regenerate^TM^ Boosting Serum (Unilever Europe, London, UK)	Calcium silicate (Ca_2_SiO_4_)+ sodium phosphate (Na_3_PO_4_) + sodium fluoride (1450ppm NaF)
SP	Sensodyne^®^ Pronamel^® ^(GlaxoSmithKline Consumer Healthcare, Surrey, UK)	Potassium nitrate (KNO_3_) + sodium fluoride (1450ppm NaF)
CD	Colgate^®^ Duraphat^®^ 5000ppm Fluoride Toothpaste (1.1% sodium fluoride) (Colgate-Palmolive, New York, NY)	1.1% sodium fluoride (5000ppm NaF)
TM	GC Tooth Mousse^TM ^(Recaldent^TM^) (GC Corporation, Tokyo, Japan)	Casein phosphopeptide (CPP) and amorphous calcium phosphate (ACP)

Enamel sample preparation

Maxillary third molars were embedded horizontally in Specifix resin (Specifix-20, Struers Ltd, Birmingham, UK). Teeth blocks were then cut into two halves (buccal and lingual) using a low-speed water-cooled microtome (Leica SP1600 Saw, Leica Instruments GmbH, Wetzlar, Germany). Surfaces of enamel were flat ground to show an area measuring 4 x 3 mm and finished sequentially with water-cooled silicon carbide discs (500 and 1200 grit, LaboPol-5, Stuers, Copenhagen, Denmark). Polishing followed using polishing cloths wet with diamond sprays (9 mm, 3 mm, and 1 mm). Afterwards, the samples were washed with distilled water in an ultrasonicator (Branson 5800, Branson Ultrasonics, Danbury, CT) for 10 minutes to remove any residues of the polishing procedure and preserved in artificial saliva until evaluation [14].

Dentine discs preparation

Maxillary third molars were embedded vertically in resin covering the whole crown of the tooth. Resin blocks were then sectioned at the middle crown level of each tooth horizontally to generate 2 mm thick discs. Dentine discs were finished, polished, and cleaned using the same sequence as enamel samples and stored in artificial saliva.

Surface microhardness testing

Before any treatment, surface microhardness (SMH) was tested for every sample. Specimens were carefully rinsed with deionized water and dried with oil-free air before SMH measurements were performed. Vickers diamond in a Wallace micro-indentation tester (H. W. Wallace & Co. Ltd., Croydon, UK) was used under a load of 250 g for 15 seconds. Each indentation was repeated six times for standardization, with an interval distance of 200 µm, starting at a point 500 µm from the edge of the enamel and dentine samples.

A second SMH registration of all samples was made after the end of the erosive-remineralization cycles. Wallace SMH readings were converted into Vickers hardness number (VHN), following calculations. The VHN is determined by the ratio F/A, where F is the force applied to the diamond and A is the surface area of the resulting indentation.

Erosion cycles

All samples, except negative control (NC), were eroded twice daily in a cola beverage (Coca-Cola; pH = 2.7) for four intervals of two minutes at 0, 12, 24, 36, 48, and 60 hours [14-17] under sustained agitation on Biometra WT 16 rocking shaker (Biometra, Göttingen, Germany), then washed with distilled water for 30 seconds utilizing a squeeze bottle. All samples were kept in paraffin-wax-stimulated natural saliva from one donor for 60 minutes before and after each erosive cycle [15,18]. After that, all test groups received their remineralization cycles for two minutes for both topical and brushing application modes, except tooth mousse, which was applied topically only for three minutes, as stated by the manufacturer’s recommendations. Artificial saliva was utilized as a lubricant storage medium for all groups. Immediately after each remineralization cycle, samples were rinsed with distilled water for 30 seconds and then kept in artificial saliva (pH 6.75), composition (g/L) (2.3 Methyl-p-hydroxybenzoate; 0.625 KCl; 0.166 CaCl2.2H2O; 1.040 K2HPO4.3H2O; 0.326 KH2PO4; 0.059 MgCl2.6H2O; 10.00 sodium carboxymethylcellulose) [19], without sorbitol [20], until the next erosive-remineralization cycle began.

Toothpaste application method

Topical Application

Toothpaste slurries were applied after each erosive challenge for two minutes [21] (three minutes for TM, as recommended by the manufacturer). Slurries of toothpaste were developed by dilution of pastes in distilled water (3:1 w/w) (0.15 ml) [19,22]. TM was applied, as supplied, without dilution (0.03 g). Finally, specimens were rinsed with distilled water for 30 seconds and stored in artificial saliva at room temperature.

Brushing Application

Slurries were brushed following the International Organization for Standardization (ISO), standard ISO/TR 14569-1:2007 [23] at 1.5N (150 gm) load pressure [24]. Two rechargeable electric multi-directional toothbrushes (Oral-B Professional Care Trizone 2000, Procter and Gamble, Cincinnati, OH) with a brushing frequency of 40,000 pulsations/min [25] were attached to metal holders against sample surfaces. Each fully charged toothbrush was fixed against a sample surface. The samples were mounted in a custom-built apparatus with silicone mould receptacles placed over a simple scale that calculated the delivered brushing load. Considering the manufacturer’s recommendations and following previous protocols [26], each sample was brushed for two minutes. After brushing, the specimens were washed with distilled water for 30 seconds and then preserved in artificial saliva at room temperature.

Specimen surface analysis

**Figure 1:**
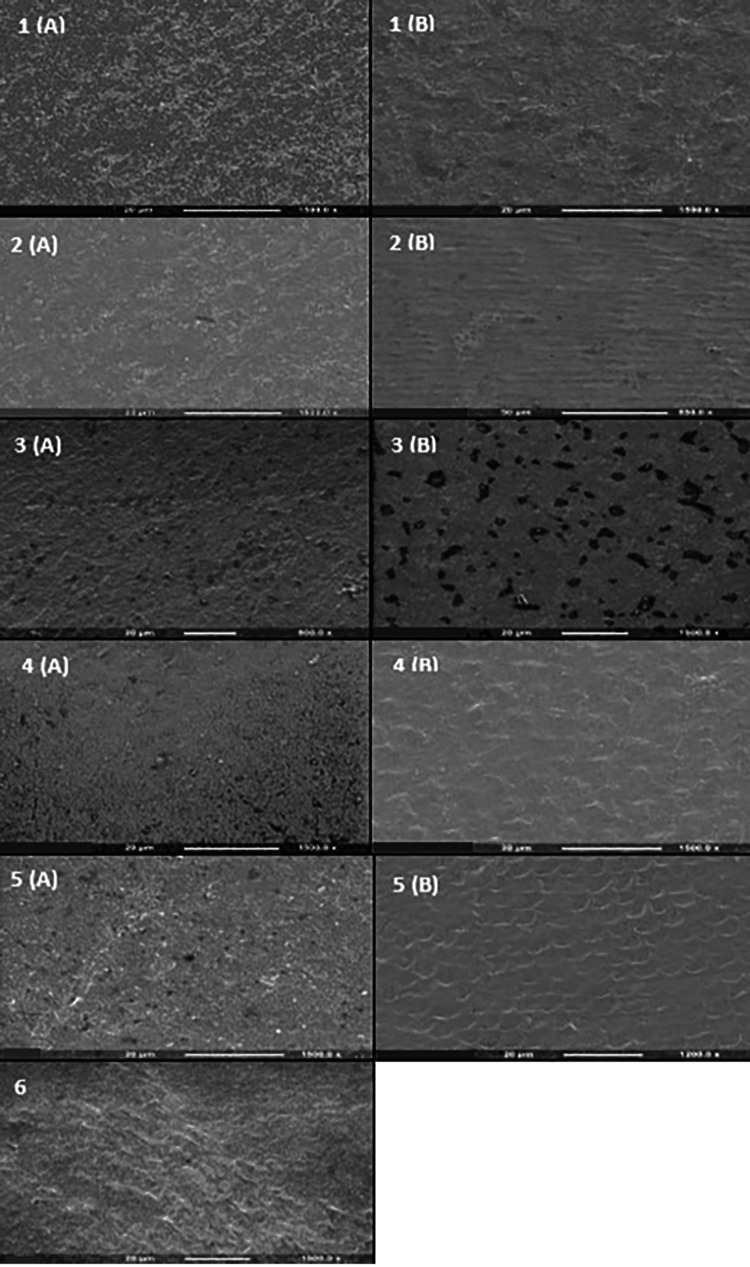
Scanning electron microscope surface images of representative samples from all enamel control and treatment groups. (1) Positive control, (2) negative control, (3) Regenerate, (4) Sensodyne Pronamel, (5) Colgate Duraphat, and (6) tooth mousse. (A) Topical application and (B) brushing application. All topical application images show a solid uniform surface covered by precipitates with no evidence of an exposed enamel prism honeycomb pattern. The surface is grainier in (A) and smoother in (B) with some evident toothbrush streaks.

**Figure 2:**
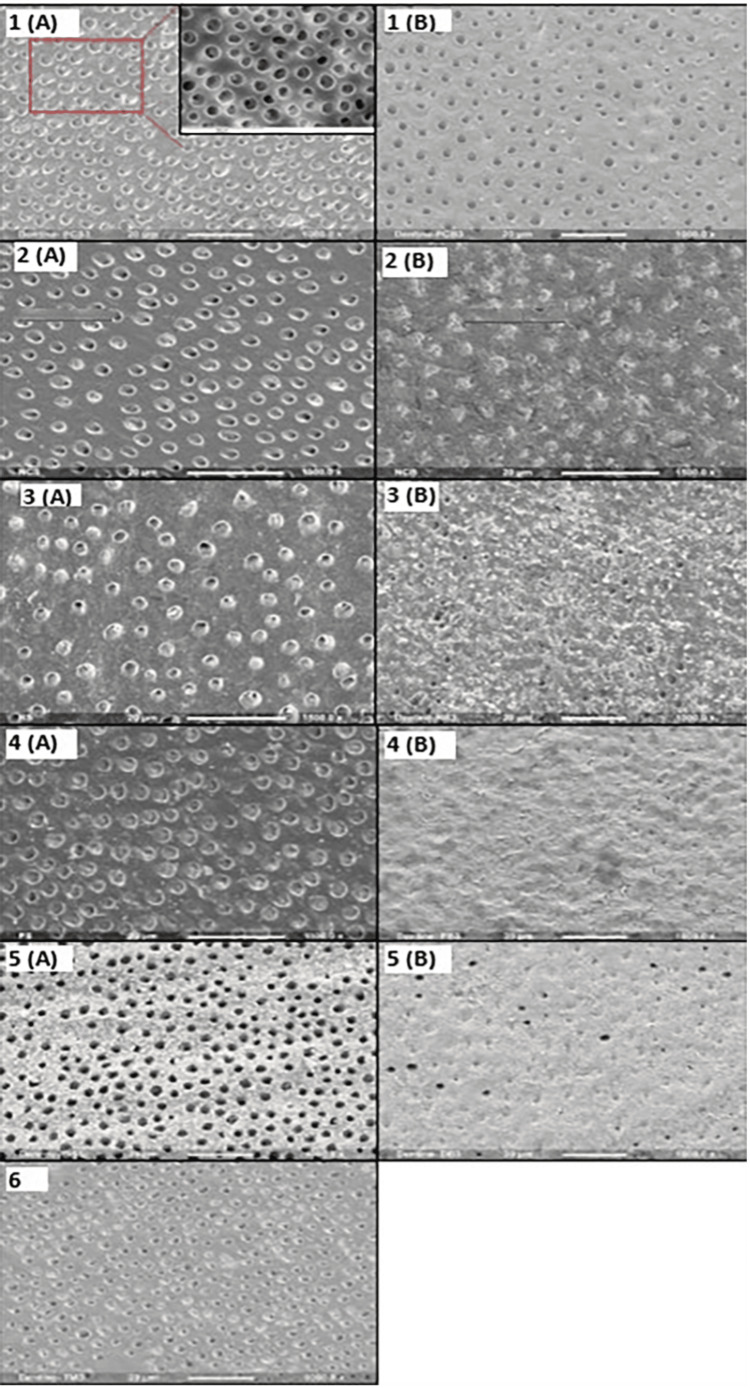
Scanning electron microscope surface images of representative samples from all dentine control and treatment groups. (1) Positive control, (2) negative control, (3) Regenerate, (4) Sensodyne Pronamel, (5) Colgate Duraphat, and (6) tooth mousse. (A) Topical application and (B) brushing application. 1 (A) Positive control topical application; hollowing and funnelling of dentinal tubules after cola erosion. Inset, a higher magnification (1500x). 1 (B) Positive control brushing application, shrinkage of dentinal tubule orifice size. 2 (A) Negative control topical application; partially filled dentinal tubules, presumably with mineral deposits from artificial saliva immersion. 2 (B) Negative control brushing application, complete blockage of dentinal tubules. 3 (A) Regenerate topical application, evident blockage of dentinal tubules by mineral deposits. 3 (B) Regenerate tooth brushing application, blockage of most dentinal tubules with over-the-surface crystal formation. 4 (A) Sensodyne Pronamel topical application, generalized blockage of dentinal tubules. 4 (B) Sensodyne Pronamel tooth brushing application, complete surface seal with toothpaste mineral deposits. 5 (A) Colgate Duraphat topical application, dentinal tubules widely open with mineral deposits between them. 5 (B) Colgate Duraphat brushing application, mineral deposits covering the surface with few open dentinal tubules. 6 Tooth mousse topical application, partial filling of dentinal tubules with mineral deposits.

Qualitative surface assessment was carried out on two representative treated samples of each group (enamel and dentine) under a scanning electron microscope (SEM; FEI, Eindhoven, Netherlands) (Figures 1, 2).

Data analysis

Enamel and dentine data were examined separately utilizing SPSS version 24 (IBM Corp., Armonk, NY). Toothpaste type and application mode variables were tested for each. Levene’s test was performed to investigate for homogeneity of the data. As the distribution was not homogeneous, a two-way ANOVA was employed using maximum likelihood estimation with robust covariance estimator, including post hoc multiple comparisons tests, applying Bonferroni corrections to compare the percentage of SMH change between groups.

## Results

The average pre- and post-treatment VHN and %SMH change of each group are plotted in Table 2. Negative %SMH reflects hardness loss while positive values reflect gain. For both enamel and dentine groups, the analysis showed significance in the %SMH change with different toothpastes, while application mode was insignificant in enamel but significant in dentine. The interaction between toothpaste and application mode was significant in both enamel and dentine tests (Tables 3, 4).

**Table 2:** Mean pre-treatment, post-treatment (VHN), and percentage of change in SMH ± standard deviation obtained in all groups. SMH: surface microhardness; VHN: Vickers hardness number.

Toothpaste group	Tooth material (Enamel/E - Dentine/D)	Application (Topical/T - Brushing/B)	Pre-treatment, mean SMH (VHN)	Post-treatment, mean SMH (VHN)	Mean SMH change (%)
Negative control (NC)	E	T	251±20	255±6	1.4±1
		B	295±22	298±4	1±0.9
	D	T	81±5	84±3	3±4
		B	82±4	88±4	8±8
Positive control (PC)	E	T	249±19	114±16	-54±13
		B	296±37	335±21	13±4
	D	T	81±3	23±2	-71±3
		B	81±2	31±3	-61±4
Regenerate (R)	E	T	261±24	181±11	-28±19
		B	302±28	186±7	-37±4
	D	T	82±3	74±2	-9±3
		B	84±4	79±11	-5±14
Regenerate + Serum (R+)	E	T	261±24	189±23	-25±18
		B	302±28	196±8	-34±4
	D	T	82±3	76±2	-6±4
		B	84±4	80±8	-3±11
Sensodyne Pronamel (SP)	E	T	260±30	223±24	-13±10
		B	300±17	192±20	-35±10
	D	T	84±3	45±10	-45±13
		B	83±4	65±11	-21±12
Colgate Duraphat (CD)	E	T	274±53	167±26	-37±24
		B	280±16	146±11	-47±2
	D	T	83±3	49±8	-41±10
		B	81±5	52±11	-35±14
Tooth Mousse (TM)	E	T	298±39	195±23	-22±9
	D	T	84±7	180±10	115±16

**Table 3:** Tests of model effects (enamel).

Source	Type III		
	Wald chi-square	df	Sig.
Toothpaste	710.726	6	0.000
Application mode	0.901	1	0.342
Toothpaste * application mode	212.375	5	0.000

**Table 4:** Tests of model effects (dentine).

Source	Type III		
	Wald chi-square	df	Sig.
Toothpaste	2929.289	6	0.000
Application mode	21.687	1	0.000
Toothpaste * application mode	11.323	5	0.055

Enamel groups

Topical Application

All topical groups decreased significantly in the SMH as compared to the negative control (NC = +1.4%, R = -28%, R+ = -25%, SP = -13%, CD = -37%, and TM = -22%). On the other hand, the positive control (PC = -54%) showed a significantly higher loss than SP (-13%), TM (-22%), R (-28%), and R+ (-25%), but not than CD (-37%). No significant differences were recorded between test groups.

Brushing Application

All brushed test groups showed a significant decrease in the SMH compared to their negative control (NC = +1%, R = -37%, R+ = -34%, SP = -35%, and CD = -47%). On the contrary, positive control showed a significant increase in SMH (PC = 13%) and that was significantly different in all groups. There was no significant variance observed between test groups except for CD, which showed a significantly higher %SMH loss than the R and R+ groups.

Topical Versus Brushing Application Modes

**Figure 3:**
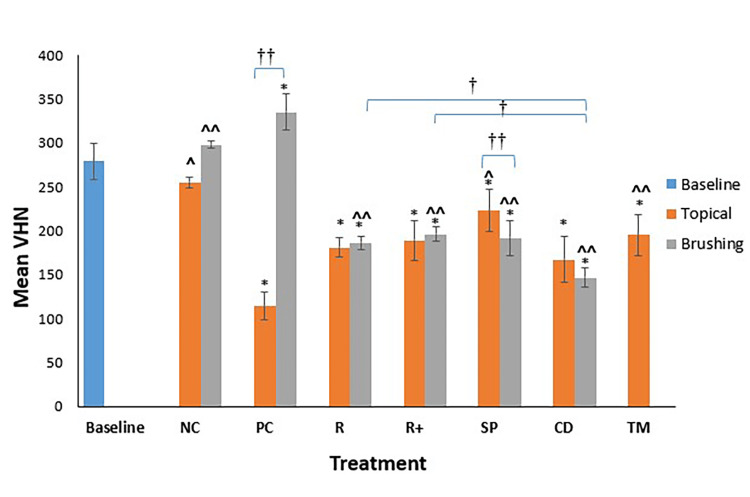
Mean VHN values for all enamel groups with all treatments at both application modes (topical and brushing). N = 8 per group. * P < 0.05, statistical difference regarding the negative control at both topical and brushing applications). ^ P < 0.01, statistical difference regarding the positive control at topical application. ^^ P < 0.01, statistical difference regarding the positive control at brushing application. ^† ^P < 0.01, statistical difference among groups. ^†† ^P < 0.05, statistical difference among topical and brushing application per group of treatment. VHN: Vickers hardness number; NC: negative control; PC: positive control; R: Regenerate; R+: Regenerate + Boosting Serum; SP: Sensodyne Pronamel; CD: Colgate Duraphat; TM: tooth mousse.

Application mode was insignificant among enamel groups. However, only topical SP and PC groups showed significant differences from their brushing counterparts (SP = -13%, -35% and PC = -54%, 13%, respectively). The mean VHN values of all enamel groups with all treatments at both application modes are presented in Figure 3.

Dentine groups

Topical Application

All topical groups had significantly decreased SMH as compared to the negative control (NC = +3%, PC = -71%, R = -9%, R+ = -6%, SP = -45%, CD = -41%, and TM = +115%). While SMH loss was significantly higher in PC than in all test groups. TM showed a significant gain in hardness (+115%) compared to all groups. Both R and R+ showed significantly lower loss than SP and CD and higher than TM. While SP and CD had comparable results.

Brushing Application

All brushed groups significantly decreased SMH as compared to the negative control, except R and R+ (NC = +8%, PC = -61%, R = -5%, R+ = -3%, SP = -21%, and CD = -35%). While SMH loss was significantly higher in PC than in all test groups. Between groups, no significance was shown, except between R, R+, and CD.

Topical Versus Brushing Application Modes

**Figure 4:**
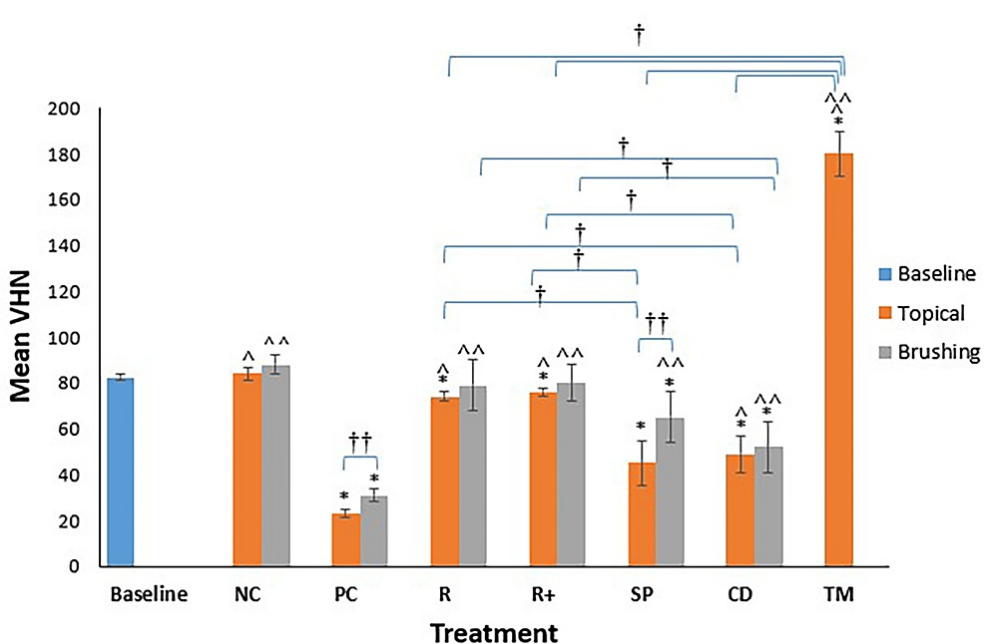
Mean VHN for all dentine groups with all treatments at both application modes (topical and brushing). N = 8 per group. * P < 0.01, statistical difference regarding the negative control at both topical and brushing applications. ^ P < 0.01, statistical difference regarding the positive control at topical application. ^^ P < 0.01, statistical difference regarding the positive control at brushing application. ^†^ P < 0.01, statistical difference among groups. ^††^ P < 0.05, statistical difference among topical and brushing application per group of treatment. VHN: Vickers hardness number; NC: negative control; PC: positive control; R: Regenerate; R+: Regenerate + Boosting Serum; SP: Sensodyne Pronamel; CD: Colgate Duraphat; TM: tooth mousse.

Application mode was significant among dentine groups. Both the SP and PC groups showed significant differences from their brushing counterparts (SP = -45%, -21% and PC = -71%, -61%, respectively), where less SMH loss was observed with brushing than with topical application. Mean VHN values of all dentine groups with all treatments at both application modes are illustrated in Figure 4 between groups.

## Discussion

In this study, both enamel and dentine specimens were exposed to erosive-remineralization cycles. Slurries of remineralizing agents were either applied topically or brushed onto the sample surface. This was done to verify the effect of daily hygiene routine on the remineralizing potential of these agents measured as percentage SMH loss or gain from a baseline reading. All agents analysed demonstrated a degree of enamel and dentine remineralization compared to the positive control.

Although profilometry is another well-established method in erosion experiments, it may be less helpful when topical agents are used. Surface microhardness was considered a sensitive tool suitable for testing different types of remineralizing agents [27,28].

The brushing load selected in this study was 150 grams (equivalent to 1.5 N), which is recommended by the ISO standard and was also found to be commonly used by healthy individuals [29]. The toothpaste application mode was statistically insignificant between enamel groups. However, topical application recorded higher SMH values than its brushing counterparts, except for the PC group. This could be related to the effect of the lesion depth on remineralizing agent diffusion. When brushing enamel, softened enamel prisms may be crushed, reducing the surface available for remineralization [30-32]. The same enamel crushing theory could explain the brushed (PC) results, which recorded higher SMH than both topical application and negative controls. Where brushing the softened enamel caused its complete removal and exposed a fresh layer to remineralization by artificial saliva, which resulted in high SMH readings or produced a compacted surface with high SMH.

Fluoride is a well-recognized remineralizing agent. The evidence that fluoride can reduce acid dissolution of enamel is well-described in the literature [32-35]. Furthermore, formulations containing calcium silicate, sodium phosphate salts, and fluoride provided in vitro benefits of enhanced enamel remineralization [36]. Our results showed remineralization offered by both R and R+ to be comparable to fluoridated toothpaste (SP and CD) among enamel samples.

CPP-ACP (TM) had a significant effect on remineralization in both enamel and dentine (%SMH change = -22% and +115%, respectively). In enamel, the action of CPP-ACP nano-complexes is well-documented to facilitate the formation of a crystal layer that fills the enamel interprism cavities and partially covers the prisms [19,37,38]. While, in dentine, CPP-ACP yields the formation of areas on the surface of the dentine matrix suggestive of an apatite precipitate.

In dentine samples, the R and R+ groups recorded the lowest SMH loss when topically applied. This suggests the efficiency of the calcium silicate products on dentine as in enamel [34,36]. The mode of action of a calcium-silicate containing cement in dentine was attributed to decreased dentine permeability as a result of created precipitates of microcrystals on the dentine surface and occlusion of dentinal tubules that ultimately results in enhancing the surface resistance to acids [2].

Different outcomes were observed with different toothpaste application modes on enamel (topical and brushing). Topical application produced better results than brushing. While, for dentine, toothpaste application mode was insignificant between groups. However, brushing groups recorded less SMH loss than their topical application counterparts. This could be attributed to the effect of brushing, which aids in pushing the agents into the open dentinal tubules allowing better penetration and in turn increased remineralization.

In enamel, SEM observations of all topically treated groups were limited to a uniform grainy surface covered with precipitates (Figure 1). The relevant SEM images of the brushed groups had intact smoother uniform surfaces (Figure 1), which may support the hypothesis of enamel prisms getting crushed with brushing. On the other hand, SEM analysis of the dentine surface allows a qualitative understanding of the demineralization-remineralization process. Specific characteristic morphological features are observed, such as the size and opening of tubules. Cola demineralized dentine surface in the PC group (Figure 2) showed larger dentinal tubule sizes than NC and all toothpaste groups. The remineralization process can be interpreted by the analysis of mineral precipitation inside or outside the tubules. In the corresponding SEM images of topical groups (Figure 2), it was generally observed that all remineralizing agents tend to cover the dentine surface forming randomly distributed crystals on the surface and filling the dentinal tubules to different levels, leaving only a few tubules exposed, least filled in the CD group. Brushed groups (Figure 2) were almost totally covered with a layer of deposits completely occluding dentinal tubules, except in the PC group (Figure 2). Our topical group observations agree with previous SEM findings by Poggio et al. [39].

Knowing that most tested products are toothpastes designed mainly for enamel remineralization rather than dentine, and their mode of use as per manufacturers’ recommendations is brushing (except tooth mousse and the regenerate boosting serum), the recommendation to topically apply toothpaste on enamel surface may not be optimal. However, the mechanical method of toothbrushing is the easiest method to reduce plaque levels [40] given good adherence to the recommended dental regimens. Plaque control remains the most effective means of improving and maintaining individuals’ oral health [41].

In light of the experiment's results, it was shown that even a short period of soft drink exposure can cause a reduction in the surface microhardness. It was also confirmed that all tested products significantly aided in the remineralization of cola-eroded enamel and dentine in an in vitro environment. It appears that toothpaste application before and after an erosive challenge has a favourable effect. Erosion-prone patients could be encouraged to execute this regimen to minimize erosive effects. However, it may be advisable to motivate patients to try to execute a non-abrasive cleaning regime after acid exposure [42]. Alternating application of different products or a combination of products may result in efficient management of erosion. Still, more research is needed to evaluate the influence of possible preventive and reparative measures against dental erosion in vivo.

Limitations

The major limitation of this study is that it is an in vitro study. Moreover, the results of the involved agents on remineralization of enamel and dentine were limited to three days of testing, which may not be able to reflect the full effect of the daily application of remineralizing agents. More research is still needed to evaluate the influence of possible preventive and reparative measures against dental erosion in vivo.

## Conclusions

Within the limitations of this in vitro study, all agents tested on enamel and dentine offered a degree of remineralization, thus protecting the teeth from further erosion progression compared to positive control. For enamel groups, all agents (Regenerate, Regenerate + serum, Sensodyne Pronamel, Colgate Duraphat, and tooth mousse) offered comparable protection against erosion challenge. Different observations were noted when the same agents were applied to the eroded dentine surface. Calcium silicate agents (Regenerate and Regenerate + serum) and tooth mousse offered a better action than the fluoride-only toothpaste (Sensodyne Pronamel and Colgate Duraphat). Moreover, contrasting results were observed for the method of application of agents (topical and brushing) for both enamel and dentine. Enamel favoured the topical application, while dentine showed a better response with brushing. However, the recommendation of tooth brushing remains the most effective measure in maintaining an individual’s oral health and no topical agent can outweigh the benefits of brushing. The topical application of tooth mousse (paste), as an additional measure, can substantially aid in the remineralization and protection of treated tooth surfaces.
